# Gender Differences in Transnational Brand Purchase Decision Toward Mixed Culture and Original Culture Advertisements: An fNIRS Study

**DOI:** 10.3389/fpsyg.2021.654360

**Published:** 2021-06-15

**Authors:** Lian Duan, Hui Ai, Lili Yang, Lianlian Xu, Pengfei Xu

**Affiliations:** ^1^Shenzhen Key Laboratory of Affective and Social Neuroscience, Center for Brain Disorders and Cognitive Sciences, Shenzhen University, Shenzhen, China; ^2^Center for Neuroimaging, Shenzhen Institute of Neuroscience, Shenzhen, China; ^3^Guangdong-Hong Kong-Macao Greater Bay Area Research Institute for Neuroscience and Neurotechnologies, Hong Kong, China

**Keywords:** functional near-infrared spectroscopy, culture mixing, neuromarketing, purchase decision, advertisement

## Abstract

Culture strategy is very important for transnational brand marketing. Functional near-infrared spectroscopy (fNIRS) is a promising brain imaging modality for neuromarketing research. In the present study, we used fNIRS to explore the neural correlates of consumers’ purchase decision on different cross-culture marketing strategies. Forty Chinese participants watched transnational brands and products advertised with photographs of the brands’ original culture (the original culture advertisements) and advertised with photographs of Chinese culture (the mixed culture advertisements), respectively. The behavioral results showed that the female participants showed significantly higher purchase rate when watching the original culture advertisements than the mixed culture advertisements, whereas the male participants did not show significant preference between these two types. The fNIRS results further revealed that for the female participants, watching mixed culture advertisements evoked significant positive activation in the left dorsolateral prefrontal cortex and negative activation in the medial prefrontal cortex, which was not found in the male participants. These findings suggest possible cognitive and emotional differences between men and women in purchase decision making toward different cross-culture marketing strategy. The present study also demonstrates the great potential of fNIRS in neuromarketing research.

## Introduction

Globalization has greatly promoted the international production and sales, providing new choices for global consumers and rich opportunities for transnational enterprises. Culture plays an important role in influencing the consumers’ attitude and purchase intention toward a brand (Zhou et al., 2015; Choi et al., 2020). However, for a transnational brand, the cultures between its origin country and host country are usually very different. Culture difference may bring fashion and exoticism feelings to the host consumers and promote the sale (Batra et al., 2000; Strizhakova et al., 2008). On the other hand, it may also bring barrier to convey value and concept from the marketers to the consumers. How to make the brands accepted by people in the host country of entirely different culture always challenge the transnational marketers (Theodosiou and Leonidou, 2003; Engelen and Brettel, 2011).

A popular marketing strategy to close this gap is to integrate the brand into the host country’s local culture. For example, marketers can launch advertisements that embed their products into the host country’s local famous scenic spots, legendary tales, or daily life. This strategy is expected to win the market by showing respect, friendship and sincerity to the host consumers (Wang and Lin, 2009; Wu, 2011). However, this strategy is not risk-free. Many transnational brands carry strong cultural information of their origin country, and they can even be viewed as symbols of their own culture (e.g., Apple is a cultural symbol of the Silicon Valley and American technology industry; Chanel represents the luxury culture of France). Integrating transnational brands into host culture usually generate culture mixing. Culture mixing refers to simultaneously presenting the representative symbols of different cultures in a same space (Chiu et al., 2009, 2011; Hao et al., 2016). Inappropriate culture mixing may induce people’s exclusive reaction (Chiu and Cheng, 2007) by enlarging the perception of distance between the original culture and the host culture (Chiu et al., 2009; Torelli et al., 2011), evoking negative emotional experience (Tong et al., 2011b; Wu et al., 2014; Morris et al., 2015), and/or bringing perceived cultural threat (Cheng, 2012). It is a hot field to study cross-culture marketing strategy for transnational brands (Cui et al., 2012, 2016; Torelli and Ahluwalia, 2012; Peng and Xie, 2016; Shi et al., 2016).

Neuromarketing is a fast-developing field. With the help of the brain imaging techniques, researchers can non-invasively measure the consumers’ neural activity. This technology offers marketing researchers opportunity to investigate the neural mechanism of the purchase behavior and discover subtle neural markers related to consumers’ decision-making process (Ariely and Berns, 2010). Functional near-infrared spectroscopy (fNIRS) is a novel non-invasive brain imaging technology. It measures the brain’s neural activity by monitoring the regional cerebral hemoglobin concentration changes (Boas et al., 2004). Compared with other modalities such as functional magnetic resonance imaging and electroencephalogram, fNIRS has many unique advantages such as low-cost, portable, comfortable, and insensitive to head motion (Hoshi, 2003). Moreover, fNIRS has relatively high temporal resolution and spatial resolution (Cutini et al., 2012). These advantages make fNIRS a very promising brain imaging modality. In recent years, pioneering studies have introduced fNIRS to culture and neural marketing studies (e.g., Murata et al., 2015; Kim et al., 2016; Çakir Murat et al., 2018; Krampe et al., 2018; Meyerding and Mehlhose, 2020). However, to our knowledge, few studies have utilized this approach to study cross-culture marketing strategy for transnational brands. Therefore, the present study motivated to explore the feasibility of using fNIRS to study the neural correlates of consumers’ purchase decision toward different cross-culture marketing strategies for transnational brands.

In the present study, forty Chinese participants watched two different types of advertisements of a series of transnational brands and products. One type of advertisements emphasized a brand’s original culture by presenting the brand with its original culture symbols (i.e., the original culture condition, OC). The other type of advertisements tried to integrate a brand into the host culture by presenting it with Chinese culture symbols (i.e., the mixed culture condition, MC). We measured the participants’ neural activity by using fNIRS when they were watching the advertisements, and compared the participants’ behavioral and neural responses between the two conditions. Moreover, previous studies suggested that cognitive and emotional differences widely exist between genders (Speck et al., 2000; Birditt and Fingerman, 2003; Labouvie-Vief et al., 2003; Goldstein et al., 2005). Therefore, we also examined the gender differences in the purchase decision under different conditions.

## Materials and Methods

### Participants

Forty healthy college students (21.2 ± 2.1 years of age, 20 males, and 20 females) from Shenzhen University participated in this study. All the participants were right-handed, with normal or corrected-to-normal vision, and without any history of psychiatric or neurological disorders. The participants were recruited via the campus network bulletin board system. Every participant received ¥40 CNY (about $6 USD) for the participation. All the participants gave written informed consent in accordance with the Declaration of Helsinki before the experiment. The study protocol was approved by the Institutional Review Board at Shenzhen Key Laboratory of Affective and Social Cognitive Science, Shenzhen University.

### Stimuli

Fifteen products of famous transnational brands were used in the present study (e.g., Adidas shoes, Apple mobile phones, and Chanel fragrance). Every product had two print advertisements (30 advertisements in total). Each advertisement consisted of a picture of the product, a logo of the brand and a background photograph. For one type of the advertisements (the mixed culture condition, MC), the background photograph contained significant Chinese culture symbols (e.g., the Great Wall or the Forbidden City), whereas for the other type of advertisements (the original culture condition, OC), the background photograph contained significant foreign cultural symbols (e.g., the Louvre of France or the Mount Rushmore of America). The advertisements were evaluated by another group of forty college student volunteers (20.9 ± 2.3 years of age, 20 males, and 20 females). For every advertisement, they rated how much the background photograph suited to advertise the brand and the product. For the mixed culture advertisements, they further rated how much they presented an integration of the brand and the product with Chinese culture, and for the original culture advertisements, they rated how much the advertisement presented an integration of the brand and the product with their original culture. The volunteers rated every question with a six-point scale (1 = not at all, 6 = very much; see Supplementary Material).

### Procedure

**FIGURE 1:**
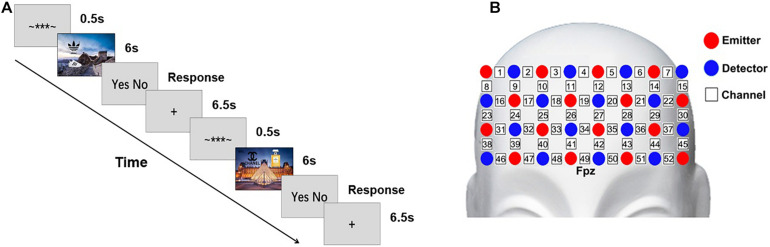
Scheme of the experiment. **(A)** The paradigm of the experiment. **(B)** The configuration of the fNIRS probes.

During the experiment, the participants were instructed to watch the advertisements and make decision that whether to buy the product shown in each advertisement. The whole procedure consisted of 30 trials. Each trial began with an indicator on the screen for 0.5 s, and then an advertisement was presented for 6 s. After the advertisement, the participants were asked to answer whether they wanted to buy the product as soon as possible by pressing a keyboard. A central fixation cross was presented for 6.5 s after the participants responded and the participants were instructed to relax and rest before the next trial. The whole procedure takes about 7 min (Figure 1A). The advertisements were presented in pseudo random sequence and counterbalanced between the two conditions.

### fNIRS Data Acquisition and Pre-processing

The fNIRS measurement was conducted with a NIRScout continuous wave fNIRS system (NIRx Medical Technologies, New York, NY, United States). A probe set containing 16 emitters and 16 detectors was placed on the frontal area, forming 52 measurement channels in total. The probe set was placed by approximately putting its bottom middle channel on Fpz of the international 10–20 system (Jasper, 1958; Figure 1B). The source-detector distance was 30 mm. The cortex localization of the optodes and channels was obtained by using the NIRSite software (NIRx Medical Technologies). The absorptions of the near-infrared lights at two wavelengths (785 nm and 830 nm) were measured with a sampling rate of 3.91 Hz. The oxygenated (HbO) and the deoxygenated (HbR) signals were calculated by using the modified Beer–Lambert law (Cope and Delpy, 1988). The differential pathlength factor were 7.25 and 6.38 for 785 nm and 830 nm, respectively (Hiraoka et al., 1993). The wavelet-based method (Duan et al., 2018) was applied to remove the superficial physiological noise in the signal. Then the signal was 0.01–0.2 Hz bandpass filtered to remove the low-frequency drift and the high-frequency noise (Zhang et al., 2017).

### fNIRS Data Analysis

The neural responses were analyzed by the general linear model approach. The regressor corresponding to each condition was generated by convolving the stimuli series with the canonical hemodynamic response function. In the individual level, the model parameters were estimated channel-by-channel for all the participants. Then the mixed effect model-based group-level activation t-maps were calculated by conducting a one-sample *t*-test on all individual parameters (Holmes and Friston, 1998; Lu et al., 2010). The activation maps of the MC condition, the OC condition, and the contrast between the two conditions (MC – OC) were generated, respectively. Only the HbO signal was used to conduct the analysis because of its high signal-to-noise ratio (Tong et al., 2011a). All the analysis were conducted with custom programmed MATLAB script.

## Results

### Behavioral Results

To validate the stimulus materials, the advertisements were rated by other volunteers than the participants engaged in the fNIRS study. For the question that how much the background photograph suited to advertise the brand and the product, all the advertisements got high average ratings, and no significant difference was observed between the MC and the OC conditions (4.93 ± 0.15 for MC and 4.90 ± 0.19 for OC, *p* = 0.66). Moreover, for the question that how much the advertisements presented an integration of the brand and the product with Chinese culture, the MC advertisements got an average rating of 4.86 ± 0.16, whereas for the question that how much the advertisements presented the original culture that the brand and the product belong to, the OC advertisements got an average rating of 4.86 ± 0.10, and no significant difference was observed between the MC and the OC advertisements (*p* = 0.96). These results suggested that both the MC and the OC advertisements had good quality in design and cultural representativeness.

**FIGURE 2:**
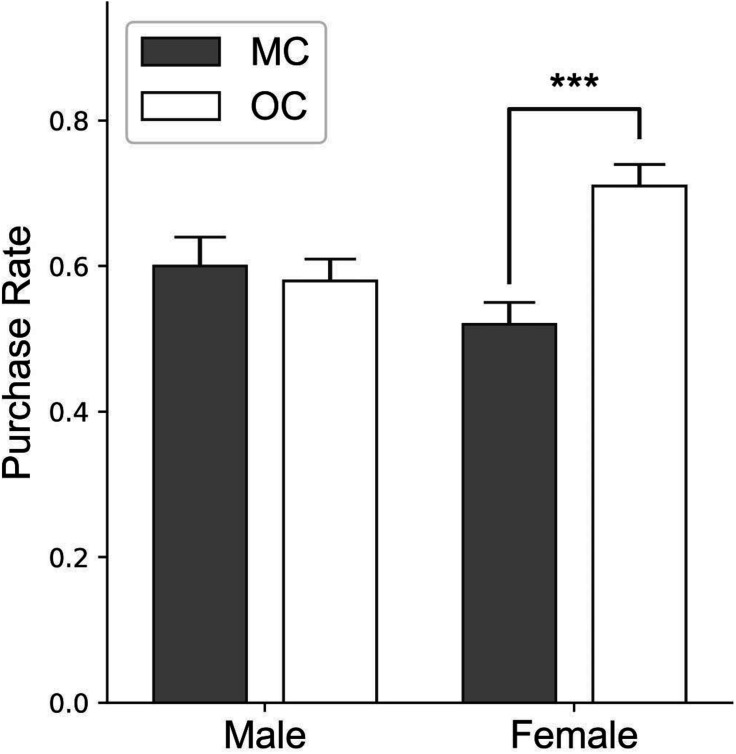
Behavioral results. The *y*-axis represents the purchase rate. Error bars denote the standard error. MC: the mixed culture condition. OC: the original culture condition. ***: *p* < 0.001.

The participants’ purchase rates were analyzed by using two-way ANOVA. Neither of the main effects (advertisement type and gender) was found significant, whereas their interaction was significant [*F*(1, 39) = 7.835, *p* = 0.007, and $\eta_{p}^{2}$ = 0.094]. The simple effect analysis revealed that for the female participants, the original culture advertisements induced significantly higher purchase rate (0.71 ± 0.16) than the mixed culture advertisements (0.52 ± 0.15, *p* = 6.98 × 10^–4^), and for the male participants, the difference between purchase rates toward the original culture advertisements (0.58 ± 0.17) and the mixed culture advertisements (0.60 ± 0.20) was insignificant (*p* = 0.72; Figure 2).

### fNIRS Results

**FIGURE 3:**
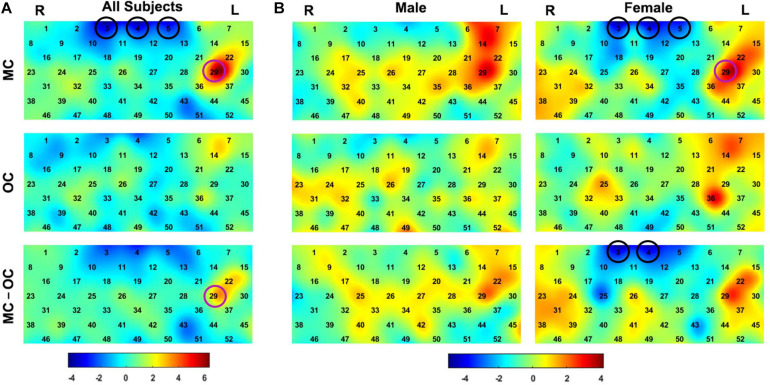
The group-level activation *t*-maps for all the participants **(A)** and for different gender **(B)**. The black circles marked the significantly negatively activated channels in mPFC, and the purple circles marked the significantly positively activated channels in dlPFC. MC: the mixed culture condition. OC: the original culture condition.

As shown in Figure 3A, the MC condition showed significant positive activation in the left dorsolateral prefrontal cortex (dlPFC) [BA 46, channel 29 (*p* = 3.62 × 10^–8^)] and negative activation in the medial prefrontal cortex (mPFC) [BA 9, channel 3 (*p* = 8.77 × 10^–6^), channel 4 (*p* = 1.31 × 10^–5^), and channel 5 (*p* = 2.10 × 10^–4^)], whereas the OC condition did not show any significant activation channels. MC showed significantly higher response than OC in the dlPFC [BA 46, channel 29 (*p* = 5.90 × 10^–4^)].

We further conducted the same analysis on the male and the female participants, respectively. As shown in Figure 3B, for the MC condition, the female participants showed significant positive activation in the left dlPFC [BA 46, channel 29 (*p* = 5.21 × 10^–4^)] and negative activation in the mPFC [BA 9, channel 3 (*p* = 1.48 × 10^–5^), channel 4 (*p* = 2.89 × 10^–5^), and channel 5 (*p* = 6.09 × 10^–4^)], and the male participants showed no significant activation. For the OC condition, neither the male nor the female participants showed significant activation. Moreover, for the female participants, MC showed significantly lower response than OC in the mPFC [BA 9, channel 3 (*p* = 2.00 × 10^–5^), and channel 4 (*p* = 4.49 × 10^–5^)]. All significances reported above were at *p* < 0.05, Bonferroni corrected.

## Discussion and Conclusion

Culture difference is an important issue in transnational marketing. In the present study, we simulated a virtual purchase scenario and measured the behavioral and neural responses toward two types of advertisements of different cross-culture marketing strategy (i.e., the mixed culture advertisement and the original culture advertisement). Behaviorally, we found that the female participants showed significantly higher purchase rate when watching the original culture advertisements than the mixed culture advertisements, whereas the male participants did not show significant preference between these two types. The fNIRS results further revealed that for the female participants, watching mixed culture advertisements evoked significant positive activation in the left dlPFC and negative activation in the mPFC, which was not found in the male participants.

In our experiment, the integration of the transnational brands with the Chinese culture symbols presented prominent culture mixing characteristics. Previous studies suggested that emotion can mediate the response to the multicultural experience (Cheng et al., 2011; Wu et al., 2014). It was also suggested that culture mixing may evoke the participants’ perception to external threats to the fidelity of in-group identity and therefore induce disgust emotion (Cheon et al., 2016). This negative emotion may further influence the cognitive information processing and the consequent decision making (Matthews and Levin, 2012). The female is generally regarded as “more emotional” than the male, and show stronger emotional physiological and neural responses than the male during emotion processing (Kring and Gordon, 1998; Labouvie-Vief et al., 2003; Hofer et al., 2006; Koch et al., 2007). Therefore, compared with the male participants, the female participants may be more sensitive to the negative influence induced by culture mixing. In the present study, we found that the female participants showed significantly lower purchase rate in the MC condition than in the OC condition, and the fNIRS results further revealed significant negative mPFC activation in the MC condition other than in the OC condition. A previous fNIRS study suggested that negative emotion stimuli could evoke negative activation in mPFC (Huang et al., 2017). Therefore, these findings indicated that the mixed culture advertisements used in the present study might induce more negative emotional experience to the female participants than the male participants, thus leading to lower purchase rate.

Cognitive fluency is another factor that may affect the response to culture mixing (Shapiro, 1999). It is suggested that individuals with high cognitive fluency precepted in processing a brand stimulus tend to make positive evaluations and *vice versa* (Lee and Labroo, 2004). It is also suggested that culture mixing may affect cognitive fluency and thus cause reduced evaluations to a brand (Torelli and Ahluwalia, 2012). In our experiment, the brand and the background photograph were culturally consistent in the OC condition. This congruency effect may facilitate the cognitive fluency. dlPFC plays critical roles in cognitive control, including receiving conflict detection signal, adjusting and reallocating cognitive resources, attention control and conflict resolving (Blais and Bunge, 2009; Bartoli et al., 2018). Interestingly, the present study found significant positive activation in the left dlPFC in the MC condition for the female participants, which may suggest that the female participants have lowered cognitive fluency when processing the mixed culture advertisements with prominent cross-culture property.

Neuromarketing approaches allow researchers to better understand various of complex purchase decision phenomena and make more comprehensive assessment of a marketing strategy, by analyzing the underlying neurobiology which are neglected or unavailable in traditional behavioral studies. The present study provided a preliminary example of using fNIRS in studying the transnational brands’ cultural marketing strategy. Our results suggested that fNIRS could effectively capture the neural responses to the cognitive and emotional processing in purchase decision making. Beyond our current experiment, fNIRS has great potential to provide experimental environment much closer to the daily life and support larger sample size of participants to assist practical transnational marketing strategy research.

This study also has several limitations that will be improved in our future works. First, the present study did not measure the participants’ emotion which may play an important role in cross-culture processing (Wu et al., 2014; Cheon et al., 2016). Second, the present study did not regard the properties of the brands and products (e.g., male-oriented or female-oriented) that may bring bias to the decisions of different gender. Third, it is necessary to use larger sample size for each gender to further confirm the current discovery.

In conclusion, the present study revealed the neural correlates of processing the original culture advertisements and the mixed culture advertisements for transnational brands. It validated the feasibility of applying fNIRS to study cross-culture marketing strategies for transnational brands and demonstrated its potential in neuromarketing research. The present study may also shed light on understanding the neural mechanism of culture mixing.

## Data Availability Statement

The raw data supporting the conclusions of this article will be made available by the authors, without undue reservation.

## Ethics Statement

The studies involving human participants were reviewed and approved by Institutional Review Board at Shenzhen Key Laboratory of Affective and Social Cognitive Science, Shenzhen University. The patients/participants provided their written informed consent to participate in this study.

## Author Contributions

LD and PX designed the research. LY and LX performed the experiments. LD and HA analyzed the data. LD drafted the work. All authors contributed to the article and approved the submitted version.

## Conflict of Interest

The authors declare that the research was conducted in the absence of any commercial or financial relationships that could be construed as a potential conflict of interest.

## References

[B1] Ariely D. Berns G. S. (2010). Neuromarketing: the hope and hype of neuroimaging in business. *Nat. Rev. Neurosci.* 11:284. 10.1038/nrn2795 20197790 PMC2875927

[B2] Bartoli E. Conner C. R. Kadipasaoglu C. M. Yellapantula S. Rollo M. J. Carter C. S. (2018). Temporal dynamics of human frontal and cingulate neural activity during conflict and cognitive control. *Cereb. Cortex (N.Y.1991).* 28 3842–3856. 10.1093/cercor/bhx245 29028974 PMC6188556

[B3] Batra R. Ramaswamy V. Alden D. L. Steenkamp J.-B. E. M. Ramachander S. (2000). Effects of brand local and nonlocal origin on consumer attitudes in developing countries. *J. Cons. Psychol.* 9 83–95. 10.1207/s15327663jcp0902_3

[B4] Birditt K. S. Fingerman K. L. (2003). Age and gender differences in adults’ descriptions of emotional reactions to interpersonal problems. *J. Gerontol. B Psychol. Sci. Soc. Sci.* 58 237–245. 10.1093/geronb/58.4.p237 12878652

[B5] Blais C. Bunge S. (2009). Behavioral and neural evidence for item-specific performance monitoring. *J. Cogn. Neurosci.* 22 2758–2767. 10.1162/jocn.2009.21365 19925177

[B6] Boas D. A. Dale A. M. Franceschini M. A. (2004). Diffuse optical imaging of brain activation: approaches to optimizing image sensitivity, resolution, and accuracy. *Neuroimage* 23 S275–S288. 15501097 10.1016/j.neuroimage.2004.07.011

[B7] Çakir Murat P. Çakar T. Girisken Y. Yurdakul D. (2018). An investigation of the neural correlates of purchase behavior through fNIRS. *Eur. J. Mark.* 52 224–243. 10.1108/ejm-12-2016-0864

[B8] Cheng C. Y. Leung K. Y. Wu T. Y. (2011). Going beyond the multicultural experience—creativity link: the mediating role of emotions. *J. Soc. Issues* 67 806–824. 10.1111/j.1540-4560.2011.01729.x

[B9] Cheng Y. Y. (2012). *Social Psychology of Globalization: JOINT Activation of Cultures and Reactions to Foreign Cultural Influence*. Ph.D. dissertation. Urbana, IL: University of Illinois at Urbana–Champaign.

[B10] Cheon B. K. Christopoulos G. I. Hong Y. Y. (2016). Disgust associated with culture mixing: why and who? *J. Cross Cult. Psychol.* 47 1268–1285. 10.1177/0022022116667845

[B11] Chiu C. Y. Cheng S. Y. Y. (2007). Toward a social psychology of culture and globalization: some social cognitive consequences of activating two cultures simultaneously. *Soc. Pers. Psychol. Comp.* 1 84–100. 10.1111/j.1751-9004.2007.00017.x

[B12] Chiu C. Y. Mallorie L. Keh H. T. Law W. (2009). Perceptions of culture in multicultural space: joint presentation of images from two cultures increases in-group attribution of culture-typical characteristics. *J. Cross Cult. Psychol.* 40 282–300. 10.1177/0022022108328912

[B13] Chiu C.y Gries P. Torelli C. J. Cheng S. Y. Y. (2011). Toward a social psychology of globalization. *J. Soc. Issues* 67 663–676. 10.1111/j.1540-4560.2011.01721.x

[B14] Choi D. W. Lee S. Alcorn M. (2020). Influence of culture on purchase decision: integrative models development of amusement park customers. *Int. J. Hosp. Manag.* 87:102502. 10.1016/j.ijhm.2020.102502

[B15] Cope M. Delpy D. T. (1988). System for long-term measurement of cerebral blood and tissue oxygenation on newborn infants by near infra-red transillumination. *Med. Biol. Eng. Comput.* 26 289–294. 10.1007/bf02447083 2855531

[B16] Cui G. Yang X. Wang H. Liu H. (2012). Culturally incongruent messages in international advertising. *Int. J. Adv.* 31:355. 10.2501/ija-31-2-355-376

[B17] Cui N. Xu L. Wang T. Qualls W. Hu Y. (2016). How does framing strategy affect evaluation of culturally mixed products? the self–other asymmetry effect. *J. Cross Cult. Psychol.* 47 1307–1320. 10.1177/0022022116670513

[B18] Cutini S. Moro S. B. Bisconti S. (2012). Review: functional near infrared optical imaging in cognitive neuroscience: an introductory review. *J. Near Infr. Spectrosc.* 20 75–92. 10.1255/jnirs.969

[B19] Duan L. Zhao Z. Lin Y. Wu X. Luo Y. Xu P. (2018). Wavelet-based method for removing global physiological noise in functional near-infrared spectroscopy. *Biomed. Opt. Exp.* 9 3805–3819. 10.1364/boe.9.003805 30338157 PMC6191612

[B20] Engelen A. Brettel M. (2011). Assessing cross-cultural marketing theory and research. *J. Bus. Res.* 64 516–523. 10.1016/j.jbusres.2010.04.008

[B21] Goldstein J. M. Jerram M. Poldrack R. Anagnoson R. Breiter H. C. Makris N. (2005). Sex differences in prefrontal cortical brain activity during fMRI of auditory verbal working memory. *Neuropsychology* 19 509–519. 10.1037/0894-4105.19.4.509 16060826

[B22] Hao J. Li D. Peng L. Peng S. Torelli C. J. (2016). Advancing our understanding of culture mixing. *J. Cross Cult. Psychol.* 47 1257–1267. 10.1177/0022022116670514

[B23] Hiraoka M. Firbank M. Essenpreis M. Cope M. Arridge S. R. Van d.Z.P, et al. (1993). A Monte Carlo investigation of optical pathlength in inhomogeneous tissue and its application to near-infrared spectroscopy. *Phys. Med. Biol.* 38:1859. 10.1088/0031-9155/38/12/011 8108489

[B24] Hofer A. Siedentopf C. M. Ischebeck A. Rettenbacher M. A. Verius M. Felber S. (2006). Gender differences in regional cerebral activity during the perception of emotion: a functional MRI study. *Neuroimage* 32 854–862. 10.1016/j.neuroimage.2006.03.053 16713306

[B25] Holmes A. P. Friston K. J. (1998). Generalisability, random effects & population inference. *Neuroimage* 7:S754.

[B26] Hoshi Y. (2003). Functional near-infrared optical imaging: utility and limitations in human brain mapping. *Psychophysiology* 40 511–520. 10.1111/1469-8986.00053 14570159

[B27] Huang Y. Mao M. Zhang Z. Zhou H. Zhao Y. Duan L. (2017). Test–retest reliability of the prefrontal response to affective pictures based on functional near-infrared spectroscopy. *J. Biomed. Opt.* 22:016011. 10.1117/1.jbo.22.1.016011 28114450

[B28] Jasper H. H. (1958). The 10–20 electrode system of the international federation. *Electr. Clin. Neurophys.* 52:10. 10590970

[B29] Kim J.-Y. Kim K.-I. Han C.-H. Lim J.-H. Im C.-H. (2016). Estimating consumers’ subjective preference using functional near infrared spectroscopy: a feasibility study. *J. Near Infr. Spectrosc.* 24 433–441. 10.1255/jnirs.1242

[B30] Koch K. Pauly K. Kellermann T. Seiferth N. Y. Reske M. Backes V. (2007). Gender differences in the cognitive control of emotion: an fMRI study. *Neuropsychologia* 45 2744–2754. 10.1016/j.neuropsychologia.2007.04.012 17544015

[B31] Krampe C. Gier N. R. Kenning P. (2018). The application of mobile fNIRS in marketing research-detecting the “first-choice-brand”. *Effect Front. Hum. Neurosci.* 12:433. 10.3389/fnhum.2018.00433 PMC6222120 30443210

[B32] Kring A. M. Gordon A. H. (1998). Sex differences in emotion: expression, experience, and physiology. *J. Pers. Soc. Psychol.* 74 686–703. 10.1037/0022-3514.74.3.686 9523412

[B33] Labouvie-Vief G. Lumley M. A. Jain E. Heinze H. (2003). Age and gender differences in cardiac reactivity and subjective emotion responses to emotional autobiographical memories. *Emotion* 3 115–126. 10.1037/1528-3542.3.2.115 12899414

[B34] Lee A. Y. Labroo A. A. (2004). The effect of conceptual and perceptual fluency on brand evaluation. *J. Mark. Res.* 41 151–165. 10.1509/jmkr.41.2.151.28665 11670861

[B35] Lu C. M. Zhang Y. J. Biswal B. B. Zang Y. F. Peng D. L. Zhu C. Z. (2010). Use of fNIRS to assess resting state functional connectivity. *J. Neurosci. Methods* 186 242–249. 10.1016/j.jneumeth.2009.11.010 19931310

[B36] Matthews M. Levin S. (2012). Testing a dual process model of prejudice: assessment of group threat perceptions and emotions. *Motiv. Emot.* 36 564–574. 10.1007/s11031-012-9280-y

[B37] Meyerding S. G. H. Mehlhose C. M. (2020). Can neuromarketing add value to the traditional marketing research? An exemplary experiment with functional near-infrared spectroscopy (fNIRS). *J. Bus. Res.* 107 172–185. 10.1016/j.jbusres.2018.10.052

[B38] Morris M. W. Chiu C. Y. Liu Z. (2015). Polycultural psychology. *Annu. Rev. Psychol.* 66:631. 10.1146/annurev-psych-010814-015001 25251481

[B39] Murata A. Park J. Kovelman I. Hu X.-S. Kitayama S. (2015). Culturally non-preferred cognitive tasks require compensatory attention: a functional near infrared spectroscopy (fNIRS) investigation. *Cult. Brain* 3 53–67. 10.1007/s40167-015-0027-y

[B40] Peng L. Xie T. (2016). Making similarity versus difference comparison affects perceptions after bicultural exposure and consumer reactions to culturally mixed products. *J. Cross Cult. Psychol.* 47 1380–1394. 10.1177/0022022116668409

[B41] Shapiro S. (1999). When an Ad’s influence is beyond our conscious control: perceptual and conceptual fluency effects caused by incidental ad exposure. *J. Cons. Res.* 26 16–36. 10.1086/209548

[B42] Shi Y. Shi J. Luo Y. L. L. Cai H. (2016). Understanding exclusionary reactions toward a foreign culture: the influence of intrusive cultural mixing on implicit intergroup bias. *J. Cross Cult. Psychol.* 47 1335–1344. 10.1177/0022022116667844

[B43] Speck O. Ernst T. Braun J. Koch C. Miller E. Chang L. (2000). Gender differences in the functional organization of the brain for working memory. *Neuroreport* 11 2581–2585. 10.1097/00001756-200008030-00046 10943726

[B44] Strizhakova Y. Coulter R. A. Price L. L. (2008). Branded products as a passport to global citizenship: perspectives from developed and developing countries. *J. Int. Mark.* 16 57–85. 10.1509/jimk.16.4.57 11670861

[B45] Theodosiou M. Leonidou L. C. (2003). Standardization versus adaptation of international marketing strategy: an integrative assessment of the empirical research. *Int. Bus. Rev.* 12 141–171. 10.1016/s0969-5931(02)00094-x

[B46] Tong Y. Hocke L. M. Frederick B. (2011a). Isolating the sources of widespread physiological fluctuations in functional near-infrared spectroscopy signals. *J. Biomed. Opt.* 16:106005. 10.1117/1.3638128 PMC3210192 22029352

[B47] Tong Y. Y. Hui P. Z. Kwan L. Peng S. (2011b). National feelings or rational dealings? the role of procedural priming on the perceptions of cross−border acquisitions. *J. Soc. Issues* 67 743–759. 10.1111/j.1540-4560.2011.01725.x

[B48] Torelli C. J. Ahluwalia R. (2012). Extending Culturally symbolic brands: a blessing or a curse? *J. Cons. Res.* 38 933–947. 10.1086/661081

[B49] Torelli C. J. Chiu C.-Y. Tam K.-p Au A. K. C. Keh H. T. (2011). Exclusionary reactions to foreign cultures: effects of simultaneous exposure to cultures in globalized space. *J. Soc. Issues* 67 716–742. 10.1111/j.1540-4560.2011.01724.x

[B50] Wang C. L. Lin X. (2009). Migration of chinese consumption values: traditions, modernization, and cultural renaissance. *J. Bus. Ethics* 88 399–409. 10.1007/s10551-009-0308-5

[B51] Wu T.-Y. (2011). “Product pleasure enhancement: cultural elements make significant difference,” in *HCI International 2011 – Posters’ Extended Abstracts. HCI 2011. Communications in Computer and Information Science*, Vol. 173 ed. Stephanidis C. (Berlin: Springer), 247–251. 10.1007/978-3-642-22098-2_50

[B52] Wu Y. Yang Y. Chiu C.-y (2014). Responses to religious norm defection: the case of Hui Chinese Muslims not following the halal diet. *Int. J. Intercult. Relat.* 39 1–8. 10.1016/j.ijintrel.2013.08.008

[B53] Zhang S. Zheng Y. Wang D. Wang L. Ma J. Zhang J. (2017). Application of a common spatial pattern-based algorithm for an fNIRS-based motor imagery brain−computer interface. *Neurosci. Lett.* 655 35–40. 10.1016/j.neulet.2017.06.044 28663052

[B54] Zhou L. Poon P. Wang H. (2015). Consumers’ reactions to global versus local advertising appeals: a test of culturally incongruent images in China. *J. Bus. Res.* 68 561–568. 10.1016/j.jbusres.2014.09.006

