# Supplementary material for: Gender Differences in Transnational Brand Purchase Decision Toward Mixed Culture and Original Culture Advertisements: An fNIRS Study
Source: Front Psychol. 2021 Jun 15;12:654360. doi: 10.3389/fpsyg.2021.654360 (PMC8226242; doi:10.3389/fpsyg.2021.654360)
Supplement: Supplementary file 1 [file Data_Sheet_1.docx]

Supplementary Material

# Advertisements and ratings

Rating Q1: How much the background photograph suits to advertise the brand and the product? (from 1 to 6, 1 = not at all, 6 = very much)

Rating Q2: (for OC) How much the advertisement presents an integration of the brand and product with the culture of its origin? (for MC) How much the advertisement presents an integration of the brand and product with the Chinese culture? (from 1 to 6, 1 = not at all, 6 = very much)

| No. | Advertisement | Rating of Q1 | Rating of Q2 |
| --- | --- | --- | --- |
| OC_1 | 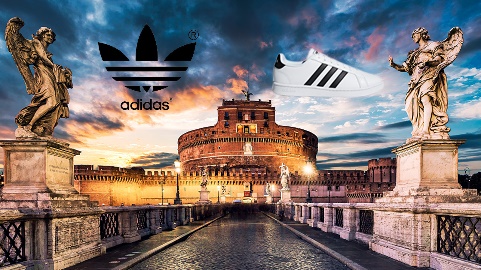 | 4.90±1.13 | 4.90±0.96 |
| MC_1 | 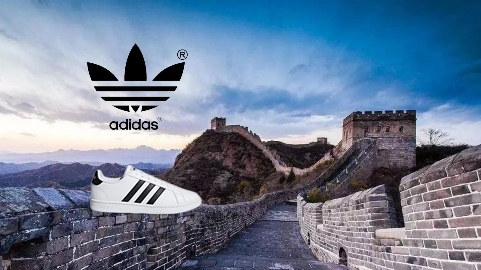 | 5.10±1.17 | 5.13±1.02 |
| OC_2 | 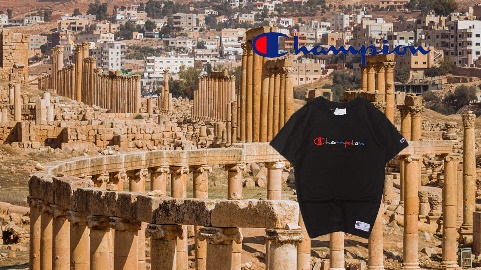 | 4.95±1.04 | 4.88±1.11 |
| MC_2 | 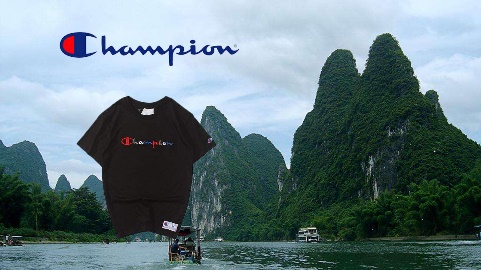 | 4.90±1.13 | 4.83±1.06 |
| OC_3 | 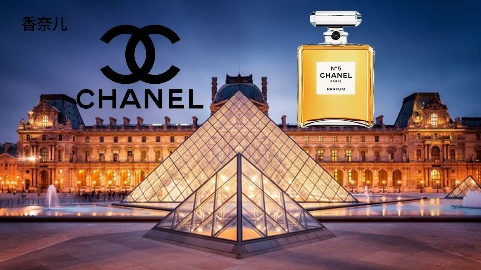 | 4.65±1.12 | 4.95±1.15 |
| MC_3 | 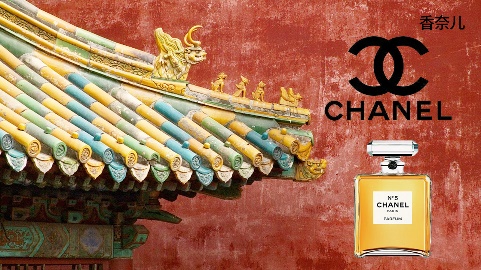 | 4.88±1.09 | 4.93±1.02 |
| OC_4 | 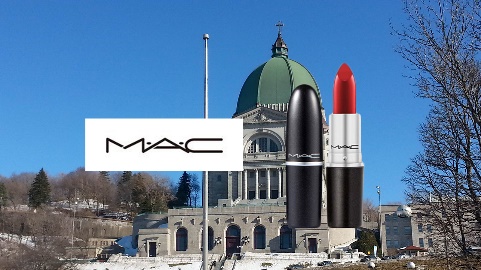 | 4.93±1.12 | 4.88±0.99 |
| MC_4 | 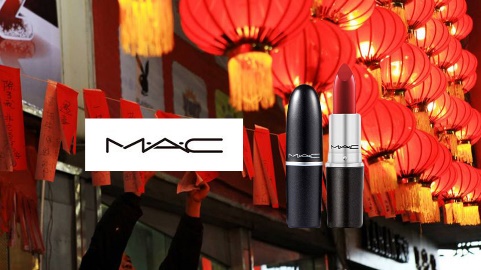 | 5.05±1.01 | 4.60±1.08 |
| OC_5 | 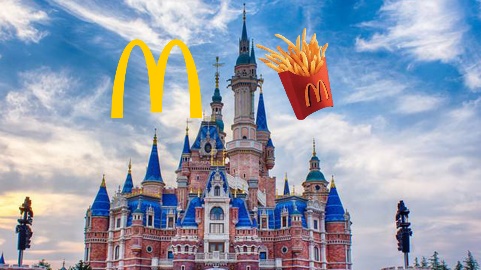 | 4.73±1.09 | 4.85±1.14 |
| MC_5 | 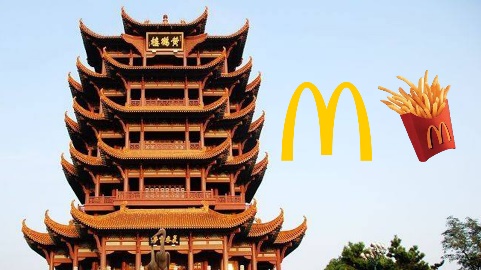 | 4.80±1.07 | 5.03±1.10 |
| OC_6 | 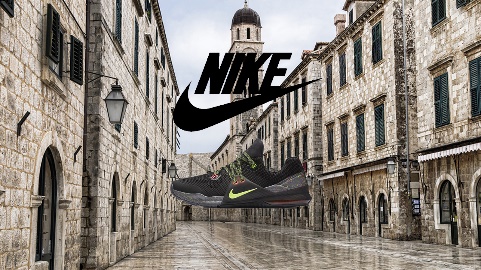 | 4.75±0.98 | 4.70±1.07 |
| MC_6 | 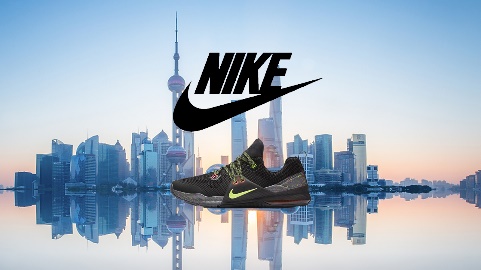 | 5.03±0.86 | 4.80±1.11 |
| OC_7 | 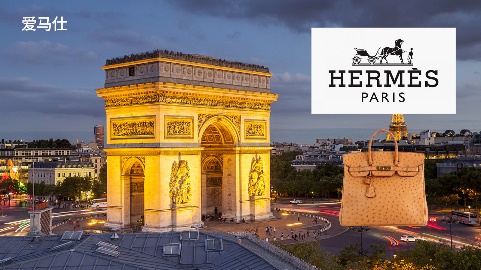 | 5.03±1.00 | 4.85±1.27 |
| MC_7 | 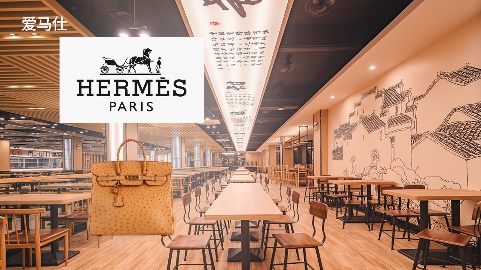 | 4.85±0.95 | 4.90±1.15 |
| OC_8 | 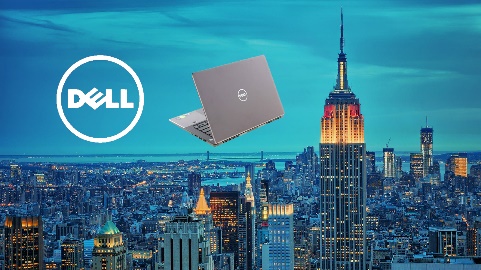 | 5.05±0.93 | 4.85±1.14 |
| MC_8 | 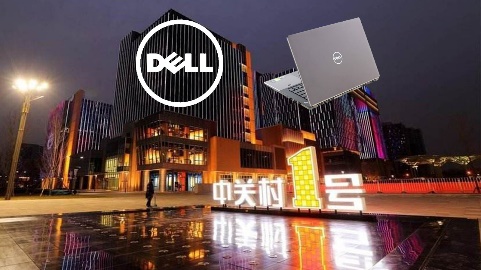 | 5.18±0.81 | 4.85±1.23 |
| OC_9 | 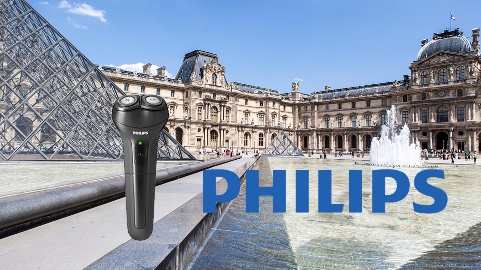 | 5.20±0.99 | 4.78±1.12 |
| MC_9 | 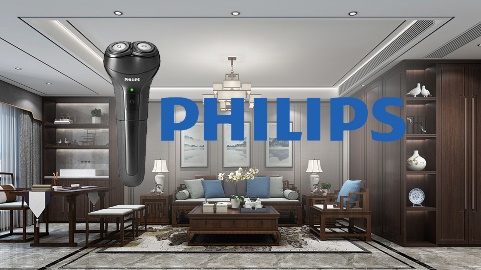 | 4.95±1.11 | 4.68±1.31 |
| OC_10 | 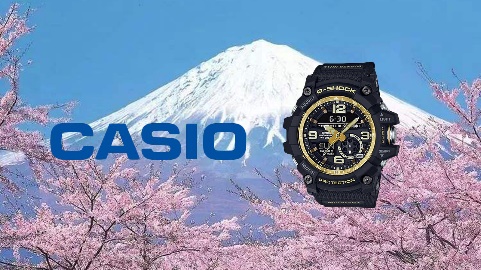 | 4.55±1.11 | 4.88±1.09 |
| MC_10 | 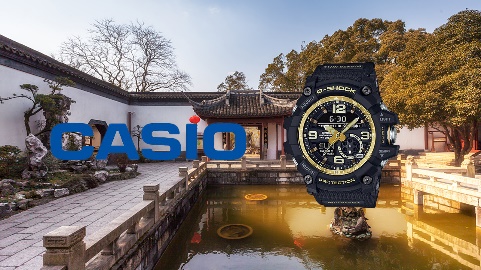 | 4.63±1.17 | 4.88±1.22 |
| OC_11 | 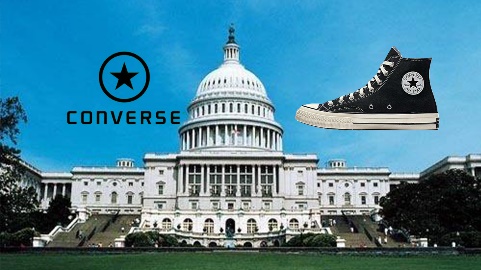 | 4.80±0.97 | 4.78±1.17 |
| MC_11 | 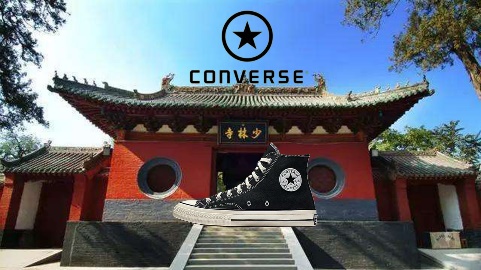 | 5.03±1.00 | 4.68±1.21 |
| OC_12 | 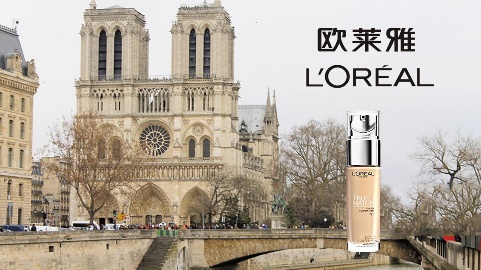 | 4.98±0.92 | 4.75±1.19 |
| MC_12 | 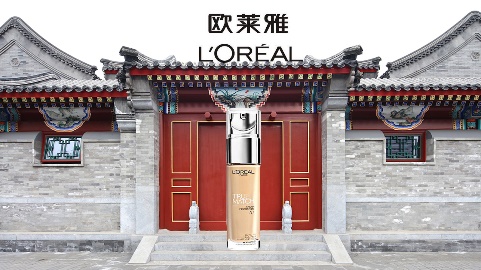 | 5.03±1.00 | 4.73±1.01 |
| OC_13 | 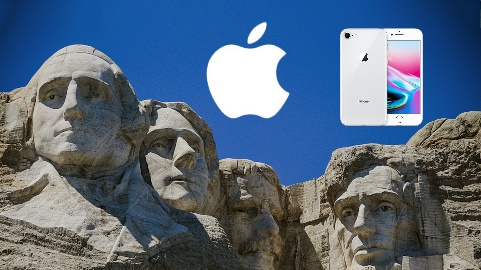 | 5.20±1.02 | 4.86±1.04 |
| MC_13 | 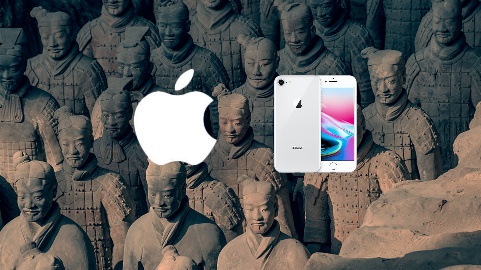 | 4.90±1.26 | 4.75±0.98 |
| OC_14 | 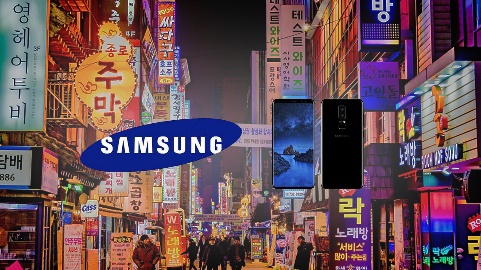 | 4.83±1.26 | 4.83±1.22 |
| MC_14 | 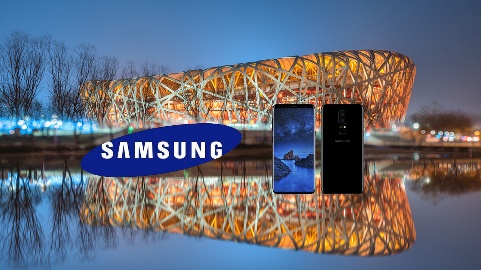 | 4.68±1.16 | 5.03±0.95 |
| OC_15 | 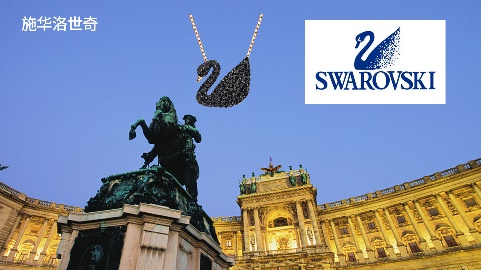 | 5.03±1.14 | 5.13±0.91 |
| MC_15 | 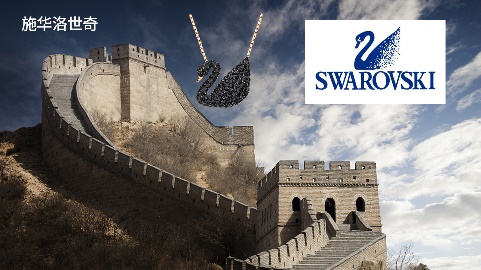 | 4.90±1.13 | 5.05±1.01 |
